# A Study on the Factors Affecting Customer Satisfaction with Institutional Foodservice during COVID-19

**DOI:** 10.3390/foods11071053

**Published:** 2022-04-06

**Authors:** Jie-Won Guak, Ji-Eun Oh, Mi-Sook Cho

**Affiliations:** 1 Department of Nutritional Science and Food Management, Ewha Womans University, Seoul 03760, Korea; jwguak@hanmail.net; 2 College of Science and Industry Convergence, Ewha Womans University, Seoul 03760, Korea; oje96@ewha.ac.kr

**Keywords:** institutional foodservice, mixed-methods design, user-based approach quality, Kano model, customer satisfaction coefficient, ISA

## Abstract

This study used a mixed-methods design combining qualitative and quantitative research to understand the factors affecting customer satisfaction with institutional foodservice during COVID-19. First, in-depth interviews and open coding were conducted with institutional foodservice users, and they indicated that harmonious menu composition, food taste, food temperature, close proximity to the restaurant, clean tableware, staff hygiene, hand sanitizer use, and table dividers were important concepts (qualities). Second, factors affecting customer satisfaction and dissatisfaction with institutional foodservice were analyzed using the Kano model, customer satisfaction coefficient, and importance–satisfaction analysis. The highest priorities derived from those analyses for improving the quality of institutional foodservice were harmonious menu composition and food temperature. This mixed-methods study is meaningful because it comprehensively analyzes the satisfaction factors important to customers of institutional foodservice, which have changed because of COVID-19. Therefore, these results will help to improve institutional foodservice and industrial development.

## 1. Introduction

Institutional foodservice provides meals to workers in corporate offices, factories, dormitories, training centers, and the like at workplace cafeterias (called staff cafeterias, staff canteens, employee canteens, staff restaurants, employee restaurants, etc.).

Institutional foodservice provides healthy, well-balanced food to workers [1,2]. Improvements in physical dining areas and the quality of employee meals increases employee satisfaction and financial performance [3,4]. Upon the introduction of institutional foodservice, productivity was improved [5], meal skipping was reduced [6], the nutritional quality of lunch was improved over a long period of time [7], and the salt content of lunch was reduced [8]. Institutional foodservice is thus an important research subject because it affects company productivity by supplying necessary nutrients to workers, which helps them maintain their health and increases work efficiency.

Institutional foodservices provide meals to many workers (i.e., customers) under established conditions and standards. Some customers are satisfied with the meal, some are dissatisfied, and some take them for granted or are indifferent. As factors of customer satisfaction with institutional foodservice, materials (location, cleanliness of dishes and cutlery, etc.), service (staff politeness, staff outfits, etc.), menu (taste and temperature of meals, variety of meals on the daily menu, etc.) [1]; variety of menu items, value for money [3]; clean and hygienic employee restaurant, various meals, delicious food [4]; and salt intake [7] have been used. As foodservice has developed, the requirements of customers who use institutional foodservice have become increasingly diverse, and in the past two years, various COVID-19-related changes have been imposed on institutional foodservice, such as social distancing and quarantine standards.

Studies of consumer perceptions and attitudes, including the dining-out environment caused by COVID-19, have been conducted. Shorter lunchtimes and social distancing are being implemented at the workplace cafeteria [9], and the importance of hand sanitizer and restaurant precautionary measures in the food supply chain and restaurant are being emphasized [10]. Safe food and a safe environment are valued above all else in self-service buffet restaurants [11], and consumers value facial recognition payment, hand sanitizer, divided dining spaces, and robot service when dining out [12]. Customers have even said they are willing to pay more to eat in restaurants that use precautionary measures [13]. In addition, food handlers in the foodservice retail sector use approved cleaning agents and disinfectants and maintain social distancing [14]. School nutrition programs emphasize the importance of public distancing, reducing touch points, and using face covers and masks for the health and safety of students and staff, along with the importance of masks, sterilization, and hygiene [15].

However, most of those studies were quantitative and measured satisfaction and revisiting intention using existing satisfaction factors. No previous study has comprehensively analyzed satisfaction factors by beginning with the quality perceived by consumers.

Garvin (1984) said that individual consumers have different wants or needs, and the goods that best satisfy their individual preferences have the highest quality for them [16]. Park (2018) said that quality is the ability to satisfy customers and provide customer satisfaction [17], and Oliver (2010) said that customer satisfaction is an individual’s perception of a product or service [18]. 

Most quality-related institutional foodservice studies have used definitions of quality from previous studies. In addition, many studies have considered customer satisfaction, customer loyalty, and revisit intention, and many studies have used the one-dimensional method, assuming that customer satisfaction increases when customer needs are met.

The Kano model, a method for researching the attributes of quality, customer needs, and customer satisfaction in quality management, industrial engineering, and business administration, has been expanded to the dining-out and bakery fields. Studies using the Kano model are lacking in the institutional foodservice field.

Therefore, this study uses a mixed-methods design to analyze customer satisfaction factors from the perspective of customers who have used institutional foodservice during COVID-19. In that way, this study aims to improve customer satisfaction and contribute to the ability of institutional foodservice to meet various consumer needs and changing food environments.

The research questions of this study are as follows.

First, how do customers perceive the quality of institutional foodservice?

Second, what are the attributes of institutional foodservice quality, and what factors affect customer satisfaction or dissatisfaction?

Third, what are the differences and improvement priorities for satisfaction with institutional foodservice quality?

## 2. Materials and Methods

### 2.1. Research Design

This study analyzed customer satisfaction factors by taking a user-based approach and using an exploratory sequential mixed method [19,20]. 

**Figure 1:**
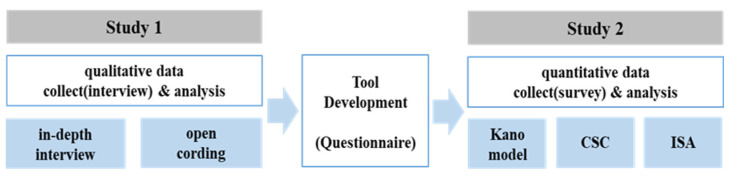
Research process.

The qualitative research method was used because it is suitable for collecting information about issues and problems in the field, focusing on the perspectives and views of various research participants, and understanding and interpreting in-depth phenomena and events experienced by research participants [21,22,23]. However, qualitative research is difficult to generalize. Therefore, the customer satisfaction factors of institutional foodservice were also comprehensively analyzed in quantitative research using data collected with a questionnaire. The research procedure is shown in Figure 1.

Study 1 was a qualitative research phase that used in-depth interviews with institutional foodservice customer to identify customer satisfaction factors and then derive quality factors for institutional foodservice derived from the qualitative research phase.

During the tool development phase, a questionnaire was written using the quality factors for institutional foodservice derived from the qualitative research phase. 

Study 2 was a quantitative research phase. After analyzing the attributes of institutional foodservice quality using the Kano model, factors affecting customer satisfaction and dissatisfaction were identified using customer satisfaction coefficient (CSC). In addition, improvement priorities for customer satisfaction were analyzed using an importance-satisfaction analysis (ISA).

### 2.2. Study 1

#### 2.2.1. Participants

This qualitative research was conducted from 8 September–13 October 2020. In this study, three adult participants who worked for a food manufacturing company, large mart, and bank, respectively, and had experience using institutional foodservices were selected as a purposive sample. Nine more people were then recruited using snowball sampling, a method of receiving recommendations from other study participants, for a total of 12 subjects.

#### 2.2.2. Measure

The in-depth interview is a method of collecting data in field studies [24] through conversations with individuals or multiple research participants [25]. 

In this study, the 12 participants were asked to describe satisfactory or unsatisfactory experiences with institutional foodservice. At this time, a semi-structured interview method was used, and an interview guide was prepared to effectively direct the in-depth interviews. The interview guide contained questions about the reasons for using institutional foodservice, satisfactory and unsatisfactory aspects of using institutional foodservice, and the factors that should be considered to further develop institutional foodservice. The interview guide focused the interviews on the study’s purpose and direction and thereby affected the expected results [26].

Open coding is the process of breaking down, examining, comparing, conceptual izing, and categorizing data by comparison [27,28]. In this study, repeated reading of the transcripts was used to derive concepts (qualities). Those qualities were then grouped by similarity, and category names were created for each category. At this time, the category names used words spoken by participants or words that researcher considered meaningful. 

To increase the reliability and validity of this study, triangulation, member checking, and peer examination were conducted. As shown in Table 1, the professor and director with experience in foodservice management and quality research conducted peer examinations, analyzed transcriptional data, and reviewed and revised the coding contents.

### 2.3. Tool Development (Questionnaire)

In Study 1, a Kano model questionnaire and ISA questionnaire were constructed using 30 qualities of institutional foodservice derived from the in-depth interviews and open coding. The Kano model questionnaire paired a positive question and a negative question for each quality, as shown in Table 2. The ISA questionnaire asked about the importance of and satisfaction with each quality, as shown in Table 3. Questions to collect demographic and foodservice usage data were added.

### 2.4. Study 2

#### 2.4.1. Participants

This quantitative research was conducted from 18 November–28 December 2020. A survey participation notice was attached to the entrance of the workplace cafeteria of the food manufacturing company, automobile manufacturer, bank, and government office where the 12 participants of the qualitative research worked. In addition, a questionnaire was distributed to adult participants who had experience using institutional foodservice and expressed their intention to participate in this study, and participants were recruited using snowball sampling. The required number of participants was calculated using previous studies and G-Power programs. A total of 390 samples were calculated as necessary, but 487 people were recruited to allow for a dropout rate of 20%. Therefore, 487 production workers, service workers, office workers, professionals, and executives completed the offline survey.

#### 2.4.2. Measure

**Figure 2:**
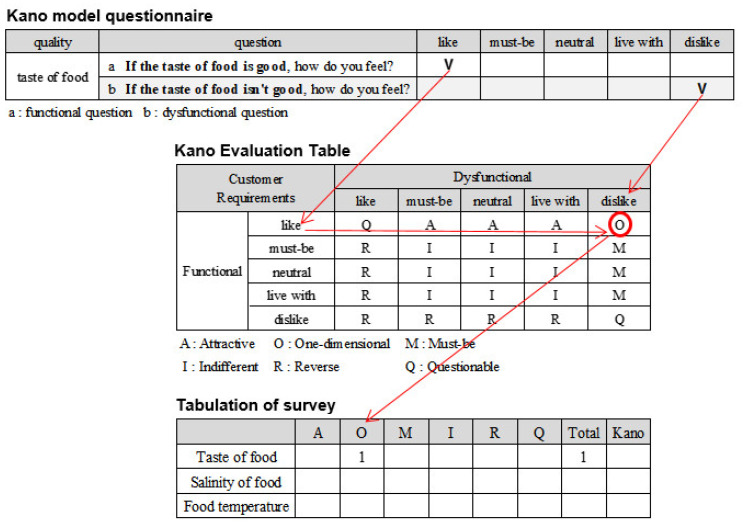
Kano evaluation table and tabulation of survey.

**Figure 3:**
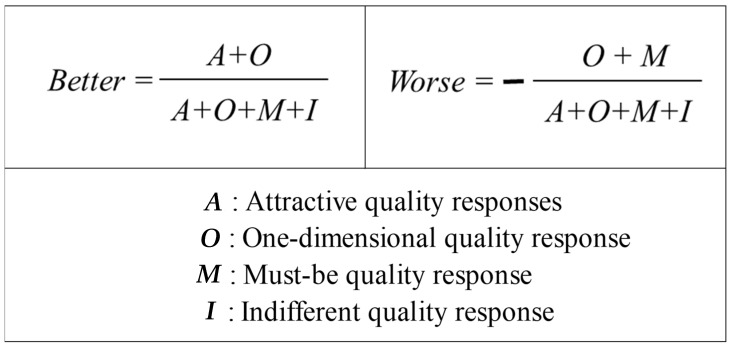
Customer satisfaction coefficient (*Better*, *Worse*).

To identify the attributes of institutional foodservice quality, a Kano evaluation table was used to classify each quality, i.e., to find the best attribute for each quality (Figure 2). A category strength of less than 6% was classified as a Combination [29]. Second, the factors affecting customer satisfaction were identified through the CSC, as shown in Figure 3, using the quality attributes classified through the Kano model.

Based on the two-factor theory [30], the Kano model classifies quality according to the degree to which a thing physically meets or does not meet the required standard in the objective aspect and the consumer’s satisfaction or dissatisfaction with the thing in the subjective aspect [31,32]. The Kano model can thus identify factors that affect customer satisfaction or dissatisfaction and is used in various industries to identify customer needs for products and services and measure customer satisfaction. It is studied in various forms and integrated with other models to form complex models [33,34,35,36,37].

The factors affecting customer satisfaction were identified through the CSC, as shown in Figure 3, using the quality attributes classified through the Kano model [38].

Third, an ISA was conducted to identify the priorities institutional foodservice improvement. The ISA theory modifies the importance–performance analysis [39] to determine improvement priorities by considering satisfaction instead of performance. First, participants completed the ISA questionnaire, which asked about the importance and satisfaction of various factors, and each response was scored and analyzed (very high = 5, high = 4, moderate = 3, low = 2, very low = 1). 

The factors tested were divided into quadrants reflecting their importance for improvement: concentrate management here (highest priority), keep up the good work, low priority for managers, and possible overkill [40].

Fourth, a nominal scale was used for demographic questions and questions about institutional foodservice usage.

All data in this study were analyzed using SPSS 22 (Statistical Package for Social Sciences Version 22.0) and Excel 10.0, and the research methods used for each analysis are shown in Table 4.

## 3. Results

### 3.1. Study 1

As shown in Table 5, in-depth interviews were conducted with 12 participants who use institutional foodservice, and open coding of their responses produced 30 qualities of interest, which were categorized into seven sub-categories and three categories, as shown in Table 6. The quality, sub-category, and category names of institutional foodservice features reflected terms used by the participants. Among the sub-categories of ingredients, hygiene, and facilities, the words ’corona’, ’cleanliness’, and ’safety’ were mentioned frequently and grouped into a category named ’safety’ [41].

Before performing this qualitative research, the quality factors for institutional foodservice reported in previous studies was examined. When the quality factors for institutional foodservice derived through the in-depth interviews and open coding in this study were compared with those reported in previous studies in Korea, most of the results were consistent, but ‘indication of the origin of the ingredients,’ ‘hand sanitizer,’ and ‘table dividers’ were not investigated in previous studies [41]. Among them, ‘hand sanitizer’ and ‘table dividers’ are concerns that have arisen with COVID-19 and can be seen as new qualities that reflect the phenomena of the times.

Thus, due to COVID-19, layout, table settings, cleanliness [42]; plexiglass partitions, hand disinfection, staff wearing disposable gloves, staff wearing protective masks or visors, disinfection of tables, cashless payment availability, disinfection of payment terminals [43]; restaurant dining environment, communication, hygiene, contactless features [44]; catering safety management, employee hygiene management, catering service, food quality, environmental atmosphere, and corporate social responsibility [45] have appeared as factors influencing customer perceptions of food-service. Similarly, in this study, hand sanitizer and plexiglass parts (table dividers) appeared, and hygiene and safety were mentioned.

The in-depth, qualitative research conducted here allowed ‘hand sanitizer’ and ‘table dividers’ to be derived as quality factors affecting institutional foodservice during COVID-19 [21].

### 3.2. Study 2

#### 3.2.1. Demographic Characteristics and Usage of Participants

For the survey, 464 (recovery rate 95.3%) questionnaires were collected after excluding those with missing or incomplete data. The demographic characteristics of the participants and the status of their institutional foodservice usage are shown in Table 7.

This study population comprised 252 men (54.3%) and 212 women (45.7%), 296 of whom were married (63.8%), and 168 of whom were unmarried (36.2%). Among the participants, the largest proportions were 30–39 years old (38.4%), university graduates (51.5%), and office workers (44.6%), and they had an average monthly income of KRW 2 million to less than KRW 3 million (42.7%).

In institutional foodservice usage, more than 10 years (28.9%), more than 5 times a week (54.3%), once a day (65.3%), lunch (60.1%), and 3–5 min (55.6%) walk time to the staff restaurants was the highest. In addition, the highest response was that the meal price was unknown (46.8%) because company pay for the meal, followed by 4000–5000 won and 5000–6000 won. The meal payment was in the order of the company (66.2%), individual (28.2%), and company-individual share burden (5.6%).

#### 3.2.2. Analysis of Institutional Foodservice Quality Attributes with the Kano Model

Customer satisfaction factors are based on customer perception of quality [31]. To identify the quality attributes important in institutional foodservice, the data from Study 1 were classified using a Kano evaluation table and CS. The quality factors of institutional foodservice were classified having the Attractive (A), One-dimensional (O), and Combination (C) attribute, as shown in Table 8.

In the meal category, ‘nutritionally balanced menu’ had a category strength (CS) of 1.7%, which had the attribute of C, a mixture of the O and A attributes. ‘Harmonious menu composition’ had a CS of 3.0%, with a C attribute, where ‘new menu, seasonal menu, favorite menu, and various type of menus, menu selectable’ had the A attribute. ‘Taste, salinity, and temperature’ all showed the O attribute.

In the service category, ‘no menu change’ was found to have the C attribute of 1.9%, as a mix of I and A attributes. ‘Adjustable Food quantity, special menu and events, and satisfaction survey and reflection of opinions’ were found to have the A attribute, and ‘friendly staff’ had the O attribute. ‘Close proximity to the restaurant and cheap meal prices’ had the A attribute, and ‘short wait time’ had the O attribute.

In the safety category, ‘fresh ingredients and good quality ingredients’ had the O attribute, and ‘indication of ingredient origin‘ had a CS of 5.6%,with the C attribute as a mixture of the O and A attributes. ‘Hand sanitizer, staff hygiene, clean tableware, clean table and clean tableware return station’ had the O attribute. ‘Hand washing station, distance between seats, and table dividers’ had the A attribute, and ‘temperature regulation’ was an O attribute.

#### 3.2.3. Analysis of Satisfaction Factors with CSC

In the Kano model, the attributes of quality are determined using the highest responses. However, if there is no difference in frequency between the highest responses and the second responses, it is difficult to evaluate customer satisfaction using only the Kano model because the attribute of the second response is ignored.

Therefore, CSC were analyzed to identify the qualities that affect customer satisfaction and increase the reliability of the results [46,47,48]. The CSC of institutional foodservice was based on responses classified in the Kano model as having the A, O, M, I attributes, and the results are shown in Table 9.

In *Better* (satisfaction coefficient), favorite menu (0.892) was the highest, followed by taste of food (0.875), special menu and events (0.868), short wait time (0.867), and close proximity to restaurant (0.853). In *Worse* (dissatisfaction coefficient), clean tableware (−0.886) was the most important, followed by clean tables (−0.847), staff hygiene (−0.767), fresh ingredients (−0.753), and taste of food (−0.700).

To increase customer satisfaction, it is obviously important to strengthen and maintain high quality in the categories that strongly affect the *Better* (satisfaction coefficient). However, because high *Worse* (dissatisfaction coefficient) scores negatively affect customer satisfaction, it is also important to improve *Worse* [38,46].

Among the attributes with high dissatisfaction coefficients, the top 12 were all O attribute, e.g., customers are satisfied if the tableware is clean and dissatisfied if it is not. Furthermore, the three attributes with the highest *Worse* are all in the hygiene category, indicating the importance of maintaining excellent hygiene. ‘Taste of food’ was ranked in the top two in *Better* and the top five in *Worse* and had an important influence on satisfaction. Therefore, ’taste of food’ is an important quality to be managed.

**Figure 4:**
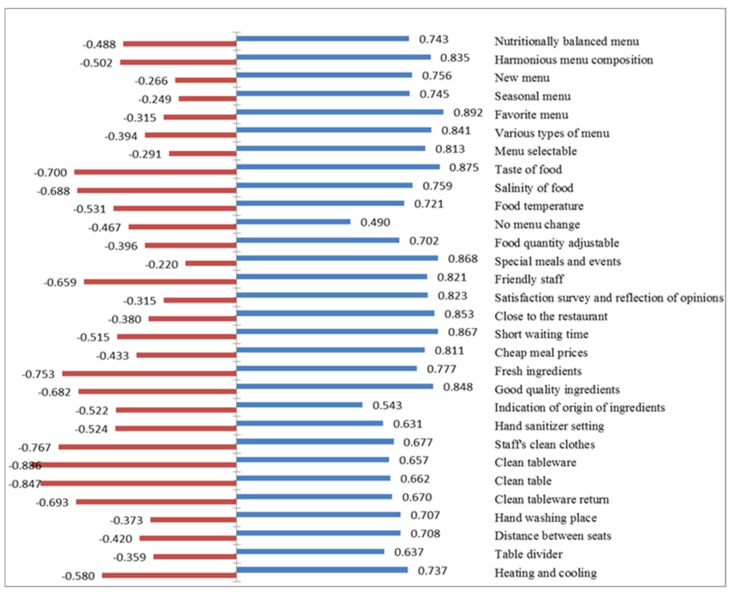
Customer Satisfaction Coefficient (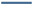: *Better*, 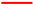: *Worse*).

The CSC is an effective way to categorize customer satisfaction factors because it can contribute to strategic decision-making about qualities to be improved and uses a distribution of continuous coefficients of *Better* and *Worse* rather than frequency [46,47,48]. Figure 4 shows the CSC *Better* (right) and *Worse* (left) for each institutional foodservice quality factor. The closer is the *Better* value is to 1, the greater the potential to increase customer satisfaction by improving or satisfying that characteristic. The closer a *Worse* value is to −1, the greater the potential to decrease customer satisfaction by failing to maintain or satisfy that characteristic.

#### 3.2.4. Importance-Satisfaction Analysis (ISA)

The Kano model can connect customer satisfaction factors with quality attributes, but it has limitations in analyzing how customer evaluate each quality attribute and indicating priorities for an improvement plan. Therefore, an ISA was conducted to provide that information.

First, a reliability analysis was performed to assess the internal consistency of the ISA questionnaire responses. As a result of calculating Cronbach’s α, as shown in Table 10, importance at 0.951, satisfaction at 0.961, and importance and satisfaction by category were all higher than 0.7.

**Figure 5:**
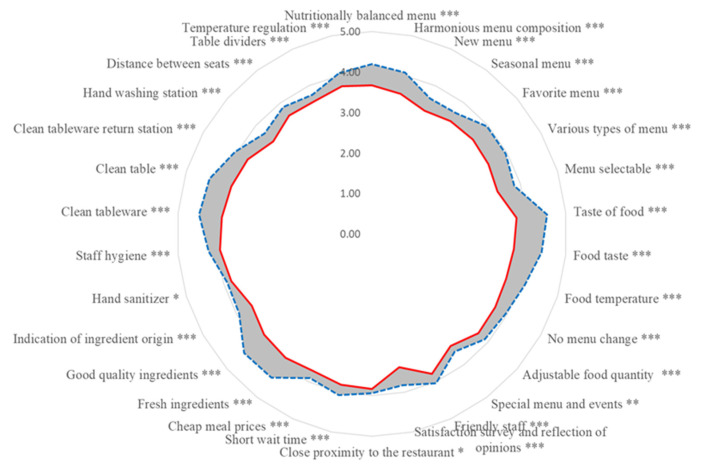
Difference between importance and satisfaction of institutional foodservice. 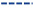 Importance, 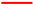 Satisfaction, * *p* < 0.05, ** *p* < 0.01, *** *p* < 0.001.

Paired *t*-test was performed to verify the difference between importance and satisfaction of institutional foodservice quality factors. As shown in Figure 5, satisfaction was lower than importance in all questions (*t* = 12.701, *p* < 0.001), and importance and satisfaction differed significantly from each other.

In these results, ‘taste of food’ had a 16.000 difference in *t*-value compared with the other quality factors for foodservice, showing the largest difference between importance and satisfaction. The *t*-value was positive (+), indicating that satisfaction was lower than importance. In ‘close proximity to the restaurant’, the difference in the *t*-value was 2.273, which was the smallest difference between importance and satisfaction. In other words, because ‘taste of food’ has a lower satisfaction value than importance value, it is important to manage the ‘taste of food’, and as shown in Table 7, it is considered that the difference between importance and satisfaction is small because the walk time is short (1 to 10 m).

**Figure 6:**
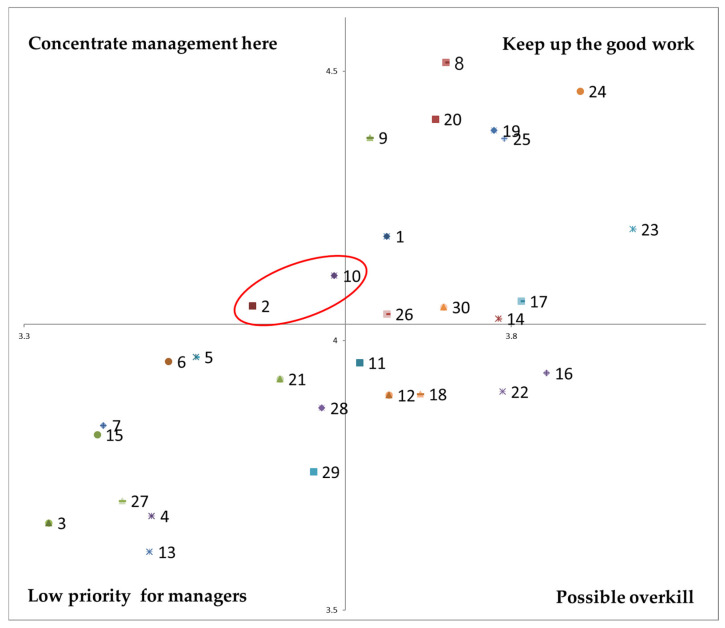
ISA of Institutional foodservice. 1. Nutritionally balanced menu; 2. Harmonious menu composition; 3. New menu; 4. Seasonal menu; 5. Favorite menu; 6. Various types of menu; 7. Menu selection; 8. Taste of food; 9. Salinity of food; 10. Food temperature; 11. No menu change; 12. Adjustable food quantity; 13. Special menu and events; 14. Friendly staff; 15. Satisfaction survey and reflection of opinions; 16. Close proximity to the restaurant; 17. Short wait time; 18. Cheap meal prices; 19. Fresh ingredients; 20. Good quality ingredients; 21. Indication of ingredient origin; 22. Hand sanitizer; 23. Staff hygiene; 24. Clean tableware; 25. Clean table; 26. Clean tableware return station; 27. Hand washing station; 28. Distance between seats; 29. Table dividers; 30. Temperature regulation.

The ISA was conducted to identify the improvement priorities for institutional foodservice. As shown in Figure 6, the qualities with the highest improvement priority (concentrate management here) were ‘harmonious menu composition’ and ‘food temperature’.

**Figure 7:**
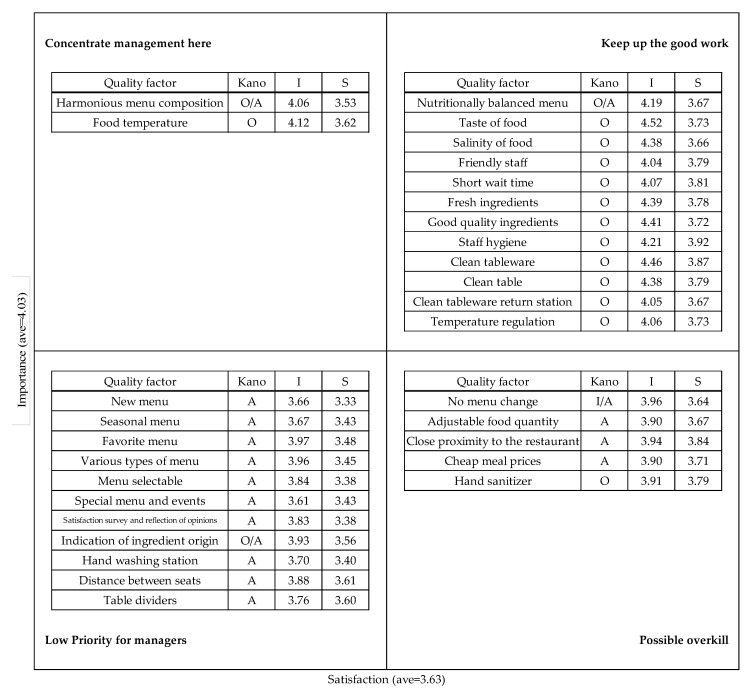
Kano-ISA of Institutional foodservice.

Figure 7 shows the Kano-ISA analysis result, which reflects the Kano model’s quality attributes and ISA.

First, ’harmonious menu composition’ and ‘food temperature’ are the most important priorities (Concentrate management here), and they have the C (O/A) and O attribute, respectively. The commonality between the two qualities is that satisfaction when conditions are met, so satisfying harmonious menu composition and food temperature is an important way to increase customer satisfaction. Therefore, preparers should pay attention to ingredients, recipes, and agreement among side dishes and work to keep warm foods warm and cold foods cold.

The ‘keep up the good work’ quadrant contains attributes with both high importance and high satisfaction. Most of the qualities in this quadrant have O attribute, so it is important to maintain and manage satisfaction.

The ‘low priority for managers’ quadrant contains attributes with low importance and satisfaction. All of those factors, except indication of ingredient origin (C:O/A), had the A attribute. ‘Indication of ingredient origin’ is considered a low priority for managers because it must be well complied by legalities. On the other hand, table dividers have already been installed in most restaurants because of COVID-19. However, even if table dividers are installed, people can be uncomfortable sitting close together, and even if table dividers are not installed, people might be comfortable sitting in a zigzag or in only one direction. Therefore, the satisfaction with the table dividers was low, which is why it is placed in the low priority for managers quadrant. In addition, ‘special menu and events’ and ‘satisfaction survey and reflection of opinion’ have the A attribute, but they are in this quadrant because they are implemented only on special day or specific day.

The qualities distributed in the ‘possible overkill’ quadrant have high satisfaction but low importance. These qualities were no menu change (C:I/A), adjustable food quantity (A), close proximity to the restaurant (A), cheap meal prices (A), and hand sanitizer (O). These qualities reflect the general characteristics of institutional foodservice, and satisfaction with these qualities is generally high. Institutional foodservice is buffet-style restaurant that can control the amount of food autonomously. Additionally, it is close to work place, and meal price is cheap, and satisfaction of hand sanitizer provided at the entrance and inside the restaurant due to COVID-19 was high. In other words, it is considered to be distributed in ‘Possible overkill’ due to the high satisfaction of these qualities.

In addition to identifying the attributes of each quality, the Kano-ISA model has the advantage of simultaneously analyzing importance, satisfaction, and priority for improvement. When satisfaction is lower than importance, the qualities appear in the ‘Concentrate management here’ quadrant for urgent improvement. The qualities distributed in ‘Keep up the good work’ and ‘Possible overkill’ can be maintained in their current state because satisfaction is higher than importance. The qualities distributed in the ‘Lower priority for managers’ quadrant have low importance and satisfaction, but improving satisfaction is still important.

## 4. Discussion

Due to COVID-19, consumers not only want nutritious food, but are also concerned about safety [49,50]. Therefore, when dining out, customers prefer a place with good preventive measures, such as table dividers to prevent splashing during meals and good ventilation [13]. Checking customer’s body temperature with a thermal imaging detector at the entrance of a restaurant [51,52], recording entry with a QR code [53], and providing hand sanitizer dispensers in the restaurant are now commonplace [12,53].

Among those changes, it was necessary to newly identify the factors that affect customer satisfaction with institutional foodservice on a daily basis. Therefore, a mixed-methods design was used here to comprehensively analyze the customer satisfaction factors in institutional foodservice from a user-based approach.

First, qualities were derived from in-depth interviews and open coding, which are qualitative user-based research methods to identify customer satisfaction factors. Most of those results were consistent with the customer satisfaction factors in previous studies [41], but ‘indication of ingredient origin’, ‘hand sanitizer’, and ‘table dividers’ were new quality factors. Among them, ‘hand sanitizer’ and ‘table dividers’ are considered to reflect changes in institutional foodservice that have emerged amid the COVID-19 pandemic.

Hand sanitizer and table dividers have been installed for customer safety during the pandemic, but they are now essentials for restaurant meals, and it is predicted that they will remain basic items in institutional foodservice.

The Kano model showed that the quality of institutional foodservice can be judged in terms of Attractive quality, One-dimensional, and Combination attributes. The CSC analysis showed that favorite menu, taste of food, and special menu and events had the highest satisfaction coefficients (*Better*), and clean tableware, clean table, and staff hygiene had the highest dissatisfaction coefficients (*Worse*). In the ISA, qualities for satisfaction being lower than importance (harmonious menu composition and food temperature) were distributed in the concentrate management here quadrant. In other words, to increase customer satisfaction with institutional foodservice, it is important to improve harmonious menu composition and food temperature.

On the other hand, due to the COVID-19, restaurants are recognized as dangerous area because of the high density of people gathering in one place [54]. Compared with the number of customers using institutional foodservice, the space for providing meals is small, and most customers use the space at specific time, so it is crowded. Therefore, to minimize the COVID risk in institutional foodservice spaces, customers can be asked to eat in a specific time interval, space between users and seats can be maximized, table dividers (partitions) can be installed, and customers can be asked to stay apart when returning tableware.

Studies related to dining out during the COVID-19 pandemic have shown that customers are willing to visit and higher costs at restaurants that have plexiglass dividers, air filters, and widely spaced tables and maintain preventive measures such as wearing masks while working [13]. Therefore, when designing a new restaurant for employees, it is important to include a hand washing area, thermal image detector, table dividers (partitions), proper seating distance, and ventilation so that employees can safe and sanitary meals.

COVID-19 has increased customer interest in food hygiene and safety. Institutional foodservice in Korea is mainly operated as a face-to-face, self-service buffet. However, due to COVID-19, kiosks have been introduced, and menu selection and payment are expanding to non-face-to-face methods. Recently, robots that move cutlery or side dishes to a serving table have been introduced. It is thought that the hygiene and safety of institutional foodservice will continue to develop through the introduction of new non-face-to-face services.

Although the COVID-19 pandemic is not over, the changes to foodservice it has caused are expected to continue even after the pandemic has ended. Therefore, institu-tional foodservice should remain focused on the taste of meals and service and take care to establish a safe environment in terms of hygiene, cleanliness, and facilities.

## 5. Conclusions

In Korea, it is estimated that 36.7% of workers use institutional foodservice. During the COVID-19 pandemic, it has been difficult to use foodservice because it has been repeatedly opened and closed by pandemic-related lockdowns [55] and concerns about use [56]. During this study period (8 September–28 December 2020), social distancing was eased from stage 2 to stage 1 and then upgraded to stage 1.5 in November and stage 2.5 in December. As the regulations related to the use of food and beverage facilities changed according to the current status of the pandemic [57], it is believed that participants changed their perceptions of the quality of institutional foodservices.

This study supplemented the shortcomings of both qualitative and quantitative research by using a mixed-methods design. First, customer satisfaction factors were derived through in-depth interviews and open coding. Then, quantitative research was conducted and analyzed using the Kano model, CSC, and ISA. The quality attributes of institutional foodservice derived from the qualitative research were analyzed using the Kano model. The factors affecting customer satisfaction and dissatisfaction were analyzed using CSC, and the improvement priority factors were found through an ISA and presented using a Kano-ISA model. Therefore, this study is meaningful because it comprehensively analyzes the factors that affect customer satisfaction by analyzing them from various angles and presenting improvement priorities for foodservice operators.

This study has the following limitations.

First, only 12 participants provided in-depth interviews, which limited the ability to collect various opinions. It would be meaningful to conduct in-depth interviews from more participants and compare the results according to the number of participants.

Second, institutional foodservice is characterized by the continuous provision of services to the same users under established conditions and standards. However, this study was conducted at a specific study point and could not examine changes in quality attributes over time. If a long-term longitudinal study had beed conducted, the life cycle of quality attributes in the Kano model would have evaluated [32].

Third, this study was conducted throughout institutional foodservice. It is also important to analyze and compare differences within institutional foodservice facilities by classifying them into manufacturing, distribution and services, government offices, and financial institutions. Such a facility-specific analysis could allow the needs of customers to be further satisfied by customizing services by facility type.

The environment of institutional foodservice changes over time. Because new qualities can arise and disappear as situations and users change, it is important to continue to pay attention to and work toward customer satisfaction.

## Figures and Tables

**Table 1:** Peer examination participants.

Fictitious Name	Gender	Age	Degree	Profession	Remark
Ha	Female	55 yrs	Ph.D.	Professor	Hospital Food manager 20 yrs
Chae	Female	44 yrs	Ph.D.	Center for Children’s foodservice Management	Director 3 yrs

**Table 2:** Kano model question.

Quality	Question		Like	Must-Be	Neutral	Live With	Dislike
taste of food	a	If the taste of food is good, how do you feel?					
	b	If the taste of food isn’t good, how do you feel?					

a: functional question b: dysfunctional question.

**Table 3:** ISA question.

Quality	Importance					Satisfaction				
	Very High	High	Moderate	Low	Very Low	Very High	High	Moderate	Low	Very Low
taste of food										

**Table 4:** Research measurement tool.

Research Method	Research Tool
Kano model	Kano Evaluation Table
	Category Strength
CSC (Customer Satisfaction Coefficient)	*Better, Worse*
ISA (Improtance-Satisfaction Analysis)	Reality analysis
	Paired *t*-test
	ISA
Geographic characteristic, Foodservice usage	Nominal scale

**Table 5:** Characteristics of the in-depth interview participants.

Interviewee	Industry	Duty	Age	Gender
A	Finance institution	Office worker	37	Female
B	Manufacturing	Office worker	26	Female
C	Manufacturing	Profession	43	Female
D	Public office	Office worker	54	Male
E	Manufacturing	Profession	39	Male
F	Distribution/Service	Service worker	53	Male
G	Manufacturing	Executive	55	Male
H	Manufacturing	Profession	27	Male
I	Manufacturing	Profession	53	Female
J	Finance institution	Office worker	39	Male
K	Public office	Office worker	39	Female
L	Manufacturing	Office worker	46	Male

**Table 6:** Quality of Institutional foodservice derived from in-depth interview and open coding.

Quality Factor		Sub-Category	Category
Nutritionally balanced menu		Menu	Meal
Harmonious menu composition			
New menu			
Seasonal menu			
Favorite menu			
Various types of menu			
Menu selectable			
Taste of food		Taste	
Salinity of food			
Food temperature			
No menu change		Service responsiveness	Service
Adjustable food quantity			
Special menu and events			
Friendly staff			
Satisfaction survey and reflection of opinions			
Close proximity to the restaurant		User convenience	
Short wait time			
Cheap meal prices			
Fresh ingredients		Food ingredients	Safety
Good quality ingredients			
Indication of ingredient origin			
Hand sanitizer		Hygiene	
Staff hygiene			
Clean tableware			
Clean table			
Clean tableware return stastion			
Hand washing station		Facility	
Distance between seats			
Table dividers			
Temperature regulation			

**Table 7:** Demographic and foodservice usage characteristic.

Demographic Characteristics		N = 464	(%)	Foodservice Usage Characteristics		N = 464	(%)
Gender	Male	252	54.3	Usage period	<1 year	58	12.5
	Female	212	45.7		1–3 year	87	18.8
Age	20–29 years	77	16.6		3–5 year	71	15.3
	30–39 years	178	38.4		5–10 year	114	24.6
	40–49 years	131	28.2		>10 year	134	28.9
	50–59 years	75	16.2	Several times a week	1	47	10.1
	≥ 60 years	3	0.6		2	47	10.1
Marital status	Married	296	63.8		3	55	11.9
	Single	168	36.2		4	63	13.6
Education level	High school	89	19.2		≥5	252	54.3
	College	71	15.3	Several times a day	1	303	65.3
	University	239	51.5		2	147	31.7
	Graduate school	42	9.1		≥3	14	3.0
	Etc.	23	5.0	Hours of use	Breakfast	3	0.6
Monthly income	< 2000 thousand won	53	11.4		Lunch	279	60.1
	2000–2999 thousand won	198	42.7		Dinner	6	1.3
	3000–3999 thousand won	87	18.8		Brackfast & Lunch	33	7.1
	4000–4999 thousand won	39	8.4		Lunch & Dinner	138	29.7
	5000–5999 thousand won	35	7.5		Etc.	5	1.1
	≥ 6000 thousand won	52	11.2	Walk time to the staff restaurant	1–2 min	135	29.1
Industry	Manufacture	239	51.5		3–5 min	258	55.6
	Distribution/Service	100	21.6		6–9 min	54	11.6
	Offices	81	17.5		≥10 min	17	3.7
	Financial institution	44	9.5	Meal price	4000–5000 won	171	36.9
Duty	Production	125	26.9		5000–6000 won	56	12.1
	Service worker	69	14.9		>6000 won	20	4.3
	Office worker	207	44.6		Unknown (Company payment)	217	46.8
	Profession	29	6.3	Meal payment	Individual	131	28.2
	Executive	28	6.0		Company	307	66.2
	Etc.	6	1.3		Company-Individual share burden	26	5.6

**Table 8:** institutional foodservice quality attributes with the Kano model.

Category	Sub-Category	Quality Factor	A	O	M	I	R	S	Total	Kano
Meal	Menu	Nutritionally balanced menu	168	176	50	69	1		464	C (O/A)
		Harmonious menu composition	186	200	32	44	1	1	464	C (O/A)
		New menu	244	106	17	96		1	464	A
		Seasonal menu	247	97	18	100	1	1	464	A
		Favorite menu	273	140	6	44	1		464	A
		Various types of menu	227	163	20	54			464	A
		Menu selectable	268	106	28	58	3	1	464	A
	Taste	Food taste	111	294	30	28		1	464	O
		Salinity of food	107	245	74	38			464	O
		Food temperature	146	188	58	71		1	464	O
Service	Service responsiveness	No menu change	119	108	108	128	1		464	C (I/A)
		Adjustable food quantity	191	132	50	87	4		464	A
		Special menu and events	309	93	9	52		1	464	A
		Friendly staff	128	252	53	30		1	464	O
		Satisfaction survey and reflection of opinions	254	128	18	64			464	A
	User convenience	Close proximity to the restaurant	235	160	16	52	1		464	A
		Short wait time	184	215	22	39	2	2	464	O
		Cheap meal prices	200	169	28	58	3	6	464	A
Safety	Food ingredient	Fresh ingredients	82	277	71	32	2		464	O
		Good quality ingredients	119	273	42	28	2		464	O
		Indication of ingredient origin	113	139	103	109			464	C (O/A)
	hygiene	Hand sanitizer	128	165	78	93			464	O
		Staff hygiene	66	248	108	42			464	O
		Clean tableware	34	271	140	19			464	O
		Clean table	44	263	130	27			464	O
		Clean tableware return stastion	90	220	101	52		1	464	O
	Facility	Hand washing station	190	138	35	101			464	A
		Distance between seats	179	148	46	89	2		464	A
		Table dividers	163	128	36	130	7		464	A
		Temperature regulation	132	210	59	63			464	O

**Table 9:** Customer satisfaction coefficient rankings (*Better* and *Worse*).

	Quality Factor	*Better*	Kano		Quality Factor	*Worse*	Kano
1	Favorite menu	0.892	A	1	Clean tableware	−0.886	O
2	Taste of food	0.875	O	2	Clean table	−0.847	O
3	Special menu and events	0.868	A	3	Staff hygiene	−0.767	O
4	Short wait time	0.867	O	4	Fresh ingredients	−0.753	O
5	Close proximity to the restaurant	0.853	A	5	Taste of food	−0.700	O
6	Good quality ingredients	0.848	O	6	Clean tableware return stastion	−0.693	O
7	Various types of menu	0.841	A	7	Salinity of food	−0.688	O
8	Harmonious menu composition	0.835	C (O/A)	8	Good quality ingredients	−0.682	O
9	Satisfaction survey and reflection of opinions	0.823	A	9	Friendly staff	−0.659	O
10	Friendly staff	0.821	O	10	Temperature regulation	−0.580	O
11	Menu selectable	0.813	A	11	Food temperature	−0.531	O
12	Cheap meal prices	0.811	A	12	Hand sanitizer	−0.524	O
13	Fresh ingredients	0.777	O	13	Indication of ingredient origin	−0.522	C (O/A)
14	Salinity of food	0.759	O	14	Short wait time	−0.515	O
15	New menu	0.756	A	15	Harmonious menu composition	−0.502	C (O/A)
16	Seasonal menu	0.745	A	16	Nutritionally balanced menu	−0.488	C (O/A)
17	Nutritionally balanced menu	0.743	C (O/A)	17	No menu change	−0.467	C (I/A)
18	Temperature regulation	0.737	O	18	Cheap meal prices	−0.433	A
19	Food temperature	0.721	O	19	Distance between seats	−0.420	A
20	Distance between seats	0.708	A	20	Adjustable food quantity	−0.396	A
21	Hand washing station	0.707	A	21	Various types of menu	−0.394	A
22	Adjustable food quantity	0.702	A	22	Close proximity to the restaurant	−0.380	A
23	Staff hygiene	0.677	O	23	Hand washing station	−0.373	A
24	Clean tableware return stastion	0.670	O	24	Table dividers	−0.359	A
25	Clean table	0.662	O	25	Favorite menu	−0.315	A
26	Clean tableware	0.657	O	26	Satisfaction survey and reflection of opinions	−0.315	A
27	Table dividers	0.637	A	27	Menu selectable	−0.291	A
28	Hand sanitizer	0.631	O	28	New menu	−0.266	A
29	Indication of ingredient origin	0.543	C (O/A)	29	Seasonal menu	−0.249	A
30	No menu change	0.490	C (I/A)	30	Special menu and events	−0.220	A

**Table 10:** Reliability analysis of importance–satisfaction by institutional foodservice.

Category	Sub-Category	Quality Factor	Cronbach’s α	
			Importance	Satisfaction
Meal	Menu	7	0.853	0.920
	Taste	3	0.761	0.876
Service	Service responsiveness	5	0.810	0.790
	User convenience	3	0.810	0.786
Safety	Food ingredient	3	0.811	0.859
	Hygiene	5	0.863	0.877
	Facility	4	0.844	0.836
	Total	30	0.951	0.961

## Data Availability

The data presented in this study are available on request from the corresponding author. The data are not publicly available due to the institutional data policy.

## References

[1] Czarniecka-Skubina E., Górska-Warsewicz H., Trafiałek J. (2020). Attitudes and Consumer Behavior toward Foods Offered in Staff Canteens. Int. J. Environ. Res. Public Health.

[2] Roos E., Sarlio-Lähteenkorva S., Lallukka T. (2004). Having lunch at a staff canteen is associated with recommended food habits. Public Health Nutr..

[3] Omar M.S., Samah N.A., Salleh N.M. (2021). The Relationship between Food Quality and Staff Satisfaction: A Case Study of Politeknik Tuanku Syed Sirajuddin Staff Cafeteria. J. Eng. Soc. Sci..

[4] Nanu L., Cobanoglu C., Yilmaz I.H. (2020). Impact of Employee Meals on Employee Satisfaction and Hotel Financial Performance: An Experimental Study. J. Hosp. Financ. Manag..

[5] Carchiolo V., Grassia M., Longheu A., Malgeri M., Mangioni G. (2021). A Network-Based Analysis of a Worksite Canteen Dataset. Big Data Cogn. Comput..

[6] Shin W.-Y., Kim J.-H. (2020). Use of workplace foodservices is associated with reduced meal skipping in Korean adult workers: A nationwide cross-sectional study. PLoS ONE.

[7] Jaworowska A., Rotaru G., Christides T. (2018). Nutritional Quality of Lunches Served in South East England Hospital Staff Canteens. Nutrients.

[8] Lassen A.D., Trolle E., Bysted A., Knuthsen P., Andersen E.W. (2018). The Salt Content of Lunch Meals Eaten at Danish Worksites. Nutrients.

[9] Nakamura M., Shirai Y., Sakuma M. (2021). Dietary Changes during the COVID-19 Pandemic: A Longitudinal Study Using Objective Sequential Diet Records from an Electronic Purchase System in a Workplace Cafeteria in Japan. Nutrients.

[10] Rizou M., Galanakis I.M., Aldawoud T.M., Galanakis C.M. (2020). Safety of foods, food supply chain and environment within the COVID-19 pandemic. Trends Food Sci. Technol..

[11] Zandonadi R., Botelho R., Maynard D., Akutsu R. (2021). Self-Service Restaurants in SARS-CoV-2 Pandemic. Encyclopedia.

[12] Zhong Y., Oh S., Moon H. (2021). What Can Drive Consumers’ Dining-Out Behavior in China and Korea during the COVID-19 Pandemic?. Sustainability.

[13] Rossetti T., Yoon S.Y., Daziano R.A. (2021). Customer Valuation of Preventive Measures by Restaurants during the COVID-19 Crisis.

[14] Shahbaz M., Bilal M., Moiz A., Zubair S., Iqbal H.M. (2020). Food Safety and COVID-19: Precautionary Measures to Limit the Spread of Coronavirus at Food Service and Retail Sector. J. Pure Appl. Microbiol..

[15] Eko L., Beechler L. (2020). Reopening Washington Schools 2020: School Nutrition Programs.

[16] Garvin D.A. (1984). What Does “Product Quality” Really Mean?. Sloan Manag. Rev..

[17] Park Y.T. (2018). Quality Management.

[18] Oliver R.L. (1980). A Cognitive Model of the Antecedents and Consequences of Satisfaction Decisions. J. Mark. Res..

[19] Creswell J.W. (2014). A Concise Introduction to Mixed Methods Research.

[20] Piccioli M. (2019). Educational research and Mixed Methods. Research designs, application perspectives, and food for thought. Studi Sulla Form..

[21] Merriam S.B., Grenier R.S. (2019). Qualitative Research in Practice Examples for Discussion and Analysis Second Edition Study Applications in Education.

[22] Bogdan R.C., Biklen S.K. (2006). Qualitative Research for Education: An Introduction to Theories and Methods.

[23] Trymell M. (2020). What is School Food Service Quality? (Part 1). Exploring Perceptions of Service Quality among Children and Food Service Professionals in Sweden.

[24] Spradley J.P. (1979). The Ethnographic Interview.

[25] Seidman I. (2013). Interviewing as Qualitative Research: A Guide for Researchers in Education and the Social Sciences.

[26] Roulston K., Conway C.M. (2014). Conducting and analyzing individual interviews. The Oxford Handbook of Qualitative Resarch in American Music Education.

[27] Strauss A., Corbin J.M. (1990). Basics of Qualitative Research: Grounded Theory Procedures and Techniques.

[28] Strauss A., Corbin J.M. (2014). Basics of Qualitative Research: Techniques and Procedures for Developing Grounded Theory.

[29] Lee M.C., Newcomb J.F. (1997). Applying the Kano methodology to meet customer requirements: NASA’s microgravity science program. Qual. Manag. J..

[30] Herzberg F., Mausner B., Snyderman B.B. (1959). The Motivation to Work.

[31] Kano N., Seraku N., Takahasi F., Tsuji S. (1984). Attractive Quality and Must-Be Quality. J. Jpn. Soc. Qual. Control.

[32] Kano N. Life cycle and creation of attractive quality. Proceedings of the 4th International Quality Management and Organizational Development Conference.

[33] Chen K.-J., Yeh T.-M., Pai F.-Y., Chen D.-F. (2018). Integrating Refined Kano Model and QFD for Service Quality Improvement in Healthy Fast-Food Chain Restaurants. Int. J. Environ. Res. Public Health.

[34] Cheng C.-C., Chang Y.-Y., Tsai M.-C., Chen C.-T., Tseng Y.-C. (2019). An evaluation instrument and strategy implications of service attributes in LOHAS restaurants. Int. J. Contemp. Hosp. Manag..

[35] Pai F.-Y., Yeh T.-M., Tang C.-Y. (2018). Classifying restaurant service quality attributes by using Kano model and IPA approach. Total Qual. Manag. Bus. Excel..

[36] Roy A.S., Bose D., Bera U.K. (2020). Assessment of residential institute foodservice using Kano categorization and importance-performance analysis. TQM J..

[37] Dewi S., Nugraha A. (2021). Quality of service evaluation based on importance performance analysis method and the kano model. J. Phys. Conf. Ser..

[38] Berger C., Blauth R., Boger D. (1993). A Special Issues on: Kano’s methods for Understanding customer defined quality. Cent. Qual. Manag. J..

[39] Martilla J.A., James J.C. (1977). Importance-Performance Analysis. J. Mark..

[40] Tonge J., Moore S.A. (2007). Importance-satisfaction analysis for marine-park hinterlands: A Western Australian case study. Tour. Manag..

[41] Guak J., Oh J.E., Cho W., Cho M.S. (2021). A Study on the Analysis of Customer Satisfaction Factors in Institutional Foodservice According to the Changes of Foodservice’s External Environment Due to Covid-19. J. Korean Soc. Food Cult..

[42] Hoang T., Suleri J. (2021). Customer behaviour in restaurants before and during COVID-19: A study in Vietnam. Res. Hosp. Manag..

[43] Czarniecka-Skubina E., Pielak M., Sałek P., Głuchowski A., Kobus-Cisowska J., Owczarek T. (2021). Use of Food Services by Consumers in the SARS-CoV-2 Pandemic. How the Eating Habits of Consumers Changed in View of the New Disease Risk Factors?. Nutrients.

[44] Jeong M., Kim K., Ma F., DiPietro R. (2021). Key factors driving customers’ restaurant dining behavior during the COVID-19 pandemic. Int. J. Contemp. Hosp. Manag..

[45] Ji Y.-G., Ko W.-H. (2022). Developing a Catering Quality Scale for University Canteens in China: From the Perspective of Food Safety. Sustainability.

[46] Kermanshachi S., Nipa T.J., Nadiri H. (2022). Service quality assessment and enhancement using Kano model. PLoS ONE.

[47] Gobbilla U., Shree A.B. Evaluation of Customer Satisfaction towards the Hospitality Industry: Using Kano Model. Proceedings of the International Conference on Research in Management & Technovation.

[48] Harijith R.G., Naduthodi H. (2017). Kano Model Customer Satisfaction Analysis of Medical Services. Int. Res. J. Eng. Technol..

[49] Marty L., de Lauzon-Guillain B., Labesse M., Nicklaus S. (2021). Food choice motives and the nutritional quality of diet during the COVID-19 lockdown in France. Appetite.

[50] Karaca A., Sah B., Orac E. (2020). Evaluation of food and beverage service spaces within the framework of COVID-19; A Case of Northern Cyprus, Walled City of Nicosia. Int. J. Sci. Technol. Res..

[51] Khan M.H., Yadav H. (2020). Sanitization During and After COVID-19 Pandemic: A Short Review. Trans. Indian Natl. Acad. Eng..

[52] Gursoy D., Chi C.G., Chi O.H. (2021). Effects of COVID 19 pandemic on restaurant and hotel customers’ sentiments towards dining out, traveling to a destination and staying at hotels. J. Hosp..

[53] Matsenko O., Kubatko O., Bardachenko V., Demchuk K. (2021). Transformation of the Restaurant Business as a Result of the COVID-19 Pandemic: Improving the Security of Service and Maintaining the Health of Human Capital. Health Econ. Manag. Review..

[54] Cyranoski D. (2020). How to Stop Restaurants Seeding Covid Infections. Nature.

[55] Fsnews 2020 Top 10 Issues with Foodservice in 2020, When We only Looked at “COVID-19”. http://www.fsnews.co.kr/news/articleView.html?idxno=40978.

[56] Asiae “Please just Eat Quietly.” Do you Know “COVID-19 Restaurant Manners?”. https://view.asiae.co.kr/article/2020120409490682473.

[57] COVID-19. https://mohw.go.kr.

